# Patient satisfaction with quality of care in public hospitals in Albania

**DOI:** 10.3389/fpubh.2022.925681

**Published:** 2022-12-22

**Authors:** Rezarta Kalaja, Marsida Krasniqi

**Affiliations:** ^1^Department of Medical Technical Sciences, University “Aleksandër Moisiu” Durrës, Durrës, Albania; ^2^Department of Ophthalmology, Catholic University Our Lady of Good Counsel, Tirana, Albania

**Keywords:** service quality, patient satisfaction, hospital health care, Albania, SERVQUAL

## Abstract

Healthcare service quality and hospitalized patient satisfaction in Albania have never truly received the necessary attention. The aim of this study is the assessment of key elements of service quality and their relationship with patients' satisfaction with receiving healthcare at public health institutions in Albania. The study examines five key dimensions of quality such as responsiveness, reliability, assurance, tangibles, and staff empathy to assess properly the healthcare quality and patient satisfaction in Albania based on the SERVQUAL instrument, which measures the differences between patient's perceptions of service quality received, and their expectations in terms of these five dimensions. A total of 800 randomly selected patients were interviewed in the main cities of Albania in public hospitals. A respective literature review was also a necessity to determine not only the appropriate methodology to be applied, together with the right quality dimensions to use but also for a better understanding of the link between service quality and patient satisfaction. The determination of the main quality factors which contribute to patient satisfaction, as well as, their order of importance, is another key aspect of the study, which finalizes with appropriate recommendations that might help quality improvement in future. The analysis shows that overall patient satisfaction is affected in public hospitals by all dimensions of service quality. Therefore, patients' perspective is extremely important in assessing healthcare service quality and should be taken into consideration by healthcare managers and policymakers in Albania, whenever significant reforms will be undertaken to improve the quality of services in this sector.

## Introduction

The health system in Albania is mainly public. The state provides most of the services related to promotion, prevention, diagnosis, and treatment (1). Although the private sector has been expanded more than pharmaceutical and dental services and some specialty diagnostic clinics, in several hospitals, those are mainly concentrated in Tirana. Several reforms have been carried out to improve and increase the efficiency of the public health sector, but even today, the healthcare system continues to remain centralized with high levels of hierarchy. The Ministry of Health and Social Protection (MHSP) is the leader and responsible for all policies and strategies related to the health system, and also for the coordination of all actors in and out of the system. Hospital management should lead in the process of building-out performance indicators and in observing the continuous use of those indicators to improve the quality of health care. To this effect, hospital managers should set up teams to develop guidelines and standards and monitor their implementation to ensure the quality of medical care services. However, despite the problems in the sector, the definition and measurement of quality indicators would indicate a trend of improvements made in both primary and hospital services. In this context, the main objective of the MHSP is continuous quality monitoring, setting parameters for its measurement, and setting up a stable service quality evaluation system, as well as increasing the motivation of service providers, which serves as key indicators in determining the level of service (1, 2).

The initial steps for assessing patient satisfaction in the healthcare sector were taken by Hulka et al. (3), starting with patient satisfaction in primary care services. Larsen et al. (4) further leads the questionnaires on patients' satisfaction by formulating an eight-scale questionnaire, to assess the overall satisfaction of patients in healthcare. They were followed by the questionnaires related to Ware et al. (5) that were introduced into healthcare planning, administration, and evaluation of healthcare services. Since then, numerous instruments for measuring the quality of care and patient satisfaction have been proposed by various authors, emphasizing more on how valuable and reliable those instruments are. Furthermore, the measurement of satisfaction also varies depending on the assumptions made about what satisfaction means and the number of attempts made to measure it (6). Satisfaction is a psychological concept, which is defined in different ways. Sometimes it is considered a judgment of individuals over any object or event, as there is an experience related to it in time. According to some theories, satisfaction is a cognitive endeavor, while other theories consider it as an emotional connection between individuals (7). Patients' expectations of healthcare providers and the health system in general play an essential role in the concept of patient satisfaction (8).

## Literature review

Patient satisfaction is considered as the degree of convergence between patients' expectations of ideal care and their perceptions of the care they receive (9). This view has been supported by many authors (10–12), who also emphasizes that satisfaction corresponds to the gap between expectations and perceived characteristics of the service (13). Abidova et al. (14) considers patient satisfaction as an indicator of service quality considering patients' personal experiences are a key element in showing their satisfaction or dissatisfaction. SERVQUAL is an instrument that has been widely used in healthcare studies to assess patients' perceptions of service quality (15–18).

The way consumers evaluate service quality in their mind is evaluated by applying the SERVQUAL scale (19) as a multifactorial instrument consisting of the below five dimensions: tangibles, reliability, responsiveness, safety, and empathy, characterized from 22 sets of questions (evaluations). This scale measures the gap between expectations and real perceptions, where half of the evaluations are processed in such a way as to measure customer expectations and 22 other evaluations are designed to measure real customer perceptions of service quality (20). Consequently, the gap will be the difference between the results of real perceptions and expectations, where a positive gap indicates that expectations have been met or exceeded, while a negative gap indicates a failure in meeting expectations. Gap results are usually analyzed as a total result to give an overall picture of each dimension. The selection of dimensions for measuring care quality is very critical, as it has a great influence on the policies chosen for the progress of healthcare.

For that reason, a very important challenge for any country is recognizing these different but logical expectations and recommending a responsible and balanced health system (21). Both Kahn et al. (22) and Lorenz et al. (23) have conducted extensive studies on the quality of healthcare, using a considerable number of indicators such as acceptability, validity, reliability, and feasibility. Similarly, Arah et al. (24) in his efforts to measure healthcare performance used 18 performance indicators, out of which 6 were related to healthcare, while the remaining were non-medical-related determinants. Ahenkan and Aduo-Adjei (25) in his study used nine performance indicators, while Zun et al. (11) and Bentum-Micah et al. (26) used five performance indicators such as tangibles, reliability, assurance, responsiveness, and empathy. Carrillat et al. (27) and Christia et al. (28) believed that the SERVQUAL instrument has the greatest interest in the field of medicine, as it has higher diagnostic values. Shafiq et al. (29), Jakupovic et al. (30), and Ko and Chou (31) stated that despite objections to the validity and reliability of SERVQUAL, its application in the field of healthcare is widely accepted. The Parasuraman model according to Peprah (32) and Endeshaw (33) is used on a large scale, as a conceptual framework for evaluating the quality of service in healthcare, as it emphasizes patients' perceptions of quality. Quality of care is important for patient outcomes, but perceptions of quality of care, which may not be relevant to actual quality, are likely to be its main determinants (34). Patient satisfaction is a commonly used indicator for measuring patient experience in healthcare (35). Crowe et al. (36) and Urden (37) independently emphasized that patient satisfaction is a cognitive assessment of service, but an emotionally influenced and consequently an individual subjective perception. Moreover, Crowe also pointed out that there is consistent evidence between different definitions, and that the most important determinants of satisfaction are part of interpersonal relationships and respective aspects of care. Patient satisfaction is one of the most important factors to determine the success of a healthcare facility (38). It has begun to be seen as part of service quality products which also affects clinical outcomes, economic measures, and quality of life (39).

Patients' expectations of healthcare providers and the health system in general play an essential role in the concept of patient satisfaction. Patients compare their experiences in this sector with their expectations of the services by allowing providers to measure their satisfaction (40). Patient experiences are a strong predictor of patient satisfaction (41). Levels of patient satisfaction vary significantly from state to state, and even in those countries which have similar health systems and respective health infrastructure, the changes were again noticeable, and 10% of the variance in patient satisfaction levels was explained by patient experiences (42).

## Methodology of the study

The research instruments used in this study are in function of achieving our main goal. They combine the primary with secondary data. The secondary data are the result of a review of extensive and contemporary literature, related to hospital service. Primary data were provided by a questionnaire conducted in 10 hospitals in different districts of the country, from September to October 2019. Some of the selected cities are Tirana with a total of 350 patients, Durrës with 150 patients followed by Fieri, Vlora, Shkodra, Lezha, Kavaja, and Kruja where in each of them 50 patients were interviewed. The reason why there was a larger selection of interviewed patients in Tirana has to do with the fact that being the capital, it has a greater concentration of private hospitals resulting in greater capacity for general and specific hospital services. The sample was distributed to the main and biggest hospitals attempting to cover the main areas of the country from north to south and Central Albania. Pilot testing was performed in advance, and the patient questionnaire was pre-tested in 20 randomly selected patients, who had just received the hospital service, to see if there were any problems or uncertainties in its completion as well as to test its reliability and validity. The result of the pilot test showed that the questionnaire questions were formulated correctly and were clearly understood. The sociodemographic characteristics were tabulated for descriptive statistics (Table 1).

**Table 1:** Sociodemographic data.

**Age**		**Gender**		**Education**		**Monthly Incomes**	
**Age**	**In %**	**Sex**	**In %**	**Level**	**In %**	**In Albanian Lek**	**In %**
18–30	11.5	Male	43.3	Primary education	35.9	< 30.000	59.7
30–45	23.4			High school	51.3	30.001–50.000	28.4
45–65	25.8	Female	56.7	University degree	10.7	50.001–70.000	8.2
> 65	39.3			Postgraduate	2.1	>70.000	3.7

Interviews were conducted through face-to-face meetings with patients on the hospital premises, but also outside them. The main conditions for the selection of respondents were the patients aged over 18 years and the duration of hospitalization to be more than 1 day. Fulfilling these criterions would make them have a clearer opinion about the service provided. Respondents were randomly selected, and the sample selection was made depending on the average number of patients, who have received hospital service during the year. The total sample size was determined using a single population proportion formula, with a 95% Cl and a 3% margin of error. The questions were formulated to be as comprehensible as possible by the patients by choosing the answer through the alternatives provided in the questionnaire which are scaled according to the Likert scale (1- very bad to 5- Very good). Referring the information of the questionnaire, its elements are grouped into factors, which are: **independent variables**, which are related to the dimensions of the quality of hospital service: Responsiveness (measured by 4 questions), Reliability (measured by 5 questions), Safety (measured by 9 questions), Tangibles (measured by 8 questions), and Empathy (measured by 5 questions). The **dependent variable** is overall satisfaction with service quality, and the **control variables** are age, gender, educational level, and income level. The value of the Alpha coefficient for the independent variables in total in this case is 0.947, so a high-reliability coefficient.

The instrument chosen for assessing service quality was SERVQUAL, which measures the difference between patients' expectations of service quality and the real assessment of that quality received. The service quality rating is then determined by calculating the difference between the ratings of customer's perceptions and expectations, according to the formula below:


$$\begin{array}{l} \text{SQ}=\text{P}-\text{E} \end{array}$$

where SQ is the overall service quality, P is the perception of service quality provided, and E is the expected service quality. A positive value of the gap indicates that they are satisfied, and a negative value indicates dissatisfaction.

Data entry and analysis were carried out using SPSS Statistics. Paired *t*-test was used to compare a significant difference in the mean score between expectation and perception of SERVQUAL dimensions to identify those with the largest SQ gap, which indicated the most critical dimension. The Chi-Square independence test was used to test whether two qualitative variables are independent of each other (Tables 2–6). Moreover, to examine the relationship of different variables with each other (Table 7), the equation of multiple linear regression and hierarchical regression are used. During the preparation of the questionnaire, special attention was paid not only to the structuring of the questions but also to their construction and adaptation in the right way so that the answers were all valid so as to achieve the main goal of the study. The reliability test was evaluated by measuring the internal consistency, through the calculation of the Crombach's Alfa Coefficient. Based on the calculations for Cronbach's Alfa coefficients for measuring the internal consistency of the questionnaire, it was concluded that their value was higher than 0.7 (allowed rate), indicating the consistency of the questionnaire.

**Table 2:** Chi-square test between the dimension of responsiveness and satisfaction for the quality of services in public hospitals.

**Chi-square tests**
	**Value**	**df**	**Asymp. Sig. (2-sided)**
Pearson chi-square	468.114	4	0.000
Likelihood ratio	357.617	4	0.000
Linear-by-linear association	334.631	1	0.000
*N* of valid cases	800		

**Table 3:** Chi-square test between the dimension of reliability and satisfaction for the quality of services in public hospitals.

**Chi-square tests**
	**Value**	**df**	**Asymp. Sig. (2-sided)**
Pearson chi-square	384.660	4	0.000
Likelihood ratio	282.763	4	0.000
Linear-by-linear association	285.237	1	0.000
*N* of valid cases	800		

**Table 4:** Chi-square test between the dimension of assurance and satisfaction for the quality of services in public hospitals.

**Chi-square tests**
	**Value**	**df**	**Asymp. Sig. (2-sided)**
Pearson chi-square	333.512	4	0.000
Likelihood ratio	232.150	4	0.000
Linear-by-linear association	242.540	1	0.000
*N* of valid cases	800		

**Table 5:** Chi-square test between the dimension of tangibles and satisfaction for the quality of services in public hospitals.

**Chi-square tests**
	**Value**	**df**	**Asymp. Sig. (2-sided)**
Pearson chi-Square	180.003	4	0.000
Likelihood ratio	182.755	4	0.000
Linear-by-linear association	156.815	1	0.000
*N* of valid cases	800		

**Table 6:** Chi-square test between the dimension of empathy and satisfaction for the quality of services in public hospitals.

**Chi-square tests**
	**Value**	**df**	**Asymp. Sig. (2-sided)**
Pearson chi-square	588.444	4	0.000
Likelihood ratio	458.916	4	0.000
Linear-by-linear association	401.767	1	0.000
*N* of valid cases	800		

**Table 7:** Correlation between independent variables (GAP) for public hospitals.

	**GAP** ** responsiveness**	**GAP** ** reliability**	**GAP** ** assurance**	**GAP** ** tangibles**	**GAP** ** empathy**
GAP responsiveness	1				
GAP reliability	0.671	1			
GAP assurance	0.508	0.476	1		
GAP tangibles	0.669	0.600	0.695	1	
GAP empathy	0.662	0.591	0.673	0.615	1

## Analyses and results

The key elements of hospital service that have been evaluated and measured for this study are responsiveness, reliability, assurance, tangibles, and empathy.

### Responsiveness

The data show that in the assessment of the readiness of hospital staff to help patients, 53% of them have a very good level. The same trend is followed by the evaluation of the swiftness with which hospital staff responds to their patients, where 77.1% evaluate this service as very well and well. Even the way of explaining the medical treatment of patients according to 67.3% of respondents is explained very well and well. The results on the time of delivery of hospital services were evaluated with high values. Thus, for 48.5% of patients, this assessment is at a very good level.

### Reliability

Regarding the evaluation of the dimension of reliability, among all the issues related to the evaluation of this dimension, the highest evaluation with 92.1% (very good and good) has the fact that hospitals store patient data without mistakes. This is followed by the alternative listed “Doctors are not in a hurry to examine patients” receiving 86.4% of the evaluation very well or well. The alternative “Hospital provides the right service since the first time it is given” has received 83.9% of positive evaluations, while the other two alternatives “Hospitals provide the service at the time they promised to do it” and “When patients have a problem, hospitals show a sincere interest in solving it” received respectfully 77.6 and 63.4% of positive evaluations.

### Assurance

This is another important criterion for which patient evaluations are as follows: The highest evaluation among all alternatives is related to security measurement “Patient data is stored applying the principle of confidentiality,” which received 92.8% of very well or well, followed by the alternative “Doctors have very good knowledge and technical skills” with 90.3% of positive evaluations from patients.

### Tangibles

Also to get a more complete evaluation of the hospital service, the evaluation of the equipment and technology used during the examinations and treatments in these hospitals was measured. Thus, according to this assessment, the option “Hospital staff have visually clean appearance” is rated higher than the others, where 88.1% of the ratings were very well and well, followed by the alternatives “Rooms are quiet” and “Rooms and the bathrooms are clean,” with 64.9 and 45.8% of positive evaluations, respectively.

### Empathy

Regarding the empathy of medical staff, the data in the table show that all the alternatives presented have received a very good rating. Thus, patients were most appreciative of the fact that “Medical staff respects the privacy of patients” evaluating it with 84.9% positively. It is followed by the alternative “Staff provides patients with personal attention” with 81.6% positive evaluation.

## Analysis of the relationship of service quality dimensions assessed according to SERVQUAL with patient satisfaction

Research question *: What are the dimensions that measure the quality of service according to SERVQUAL that affect patient satisfaction?*

Hypothesis 1

Ho: There is no significant impact of key dimensions of service quality on patient satisfaction in public hospitals.

Ha: There is a significant impact of the main dimensions of service quality on patient satisfaction in public hospitals.

Thus, in response to the research question, which assesses the relationship between service quality factors according to SERVQUAL and patient satisfaction, the analysis of a multiple linear regression equation is used. Consequently, the dimensions of the quality of services in public hospitals are analyzed, considering the GAP for each of them. Initially, the factor weights of the dimensions were tested according to the respective questions together with the reliability coefficients, and all the factor weights for each dimension resulted above the allowed values as given below.

To this effect, all these dimensions are kept in the analysis. At the same time, the multicollinearity between them was evaluated and all values were within the allowed norms (Table 8).

**Table 8:** Factor weights for each dimension after measuring the GAP.

**Dimension**	**Factor weights**
Responsiveness	0.915
Reliability	0.964
Assurance	0.966
Tangibles	0.938
Empathy	0.975

Value of Sig. = 0.000 according to the ANOVA analysis means that the relationship between satisfaction and quality dimensions is statistically significant. The general form of the regression equation linking GAP measured for each dimension in public hospitals and patient satisfaction results in:

(Patient satisfaction with the quality of service in public hospitals) = 3.722 + 0.247 (responsiveness) + 0.578 (reliability) + 0.130 (assurance) + 0.151 (tangibles) + 0.371 (empathy)

As can be seen from the equation, the highest value of the coefficients β-ta is observed in the dimension of reliability (Table 9), meaning that if 1 unit of this dimension is increased, the value of satisfaction increases by 0.578 times. This dimension is followed by the dimensions of empathy and responsiveness which are ranked, respectively, with coefficients of 0.371 and 0.247, followed by the tangibles and assurance dimensions. The last two dimensions are ranked in these positions because of the importance their impact on the overall satisfaction of service quality, as according to patients, the responsiveness and reliability of medical staff have a greater effect on them and in increasing their satisfaction. The table below also shows that this equation has a determinability coefficient of *R*^2^ adjusted = 51.4%, thus it determines 51.4% of the change in the values of the variance of the patient satisfaction level (Table 10).

**Table 9:** Regression coefficients on the GAP for public hospitals.

**Model**	**Unstandardized coefficients**		**Standardized coefficients**	***t***	**Sig**.
	**B**	**Std. Error**	**Beta**		
Responsiveness	3.722	0.032		114.606	0.000
Reliability	0.247	0.045	0.352	5.509	**0.000**
Assurance	0.578	0.070	−0.562	−8.304	**0.000**
Tangibles	0.130	0.059	0.153	2.212	**0.027**
Empathy	0.151	0.047	0.143	3.194	**0.001**
Responsiveness	0.371	0.055	0.523	6.785	**0.000**

Highlighted sigma values show statistically significant dimensions.

**Table 10:** Multiple regression analysis between GAP-s of independent variables and dependent variable patient satisfaction from services in public hospitals.

**Model**	***R* ^2^**	***R*^2^ adjusted**	***t***	**Sig**.
Constant	0.0519	**0.514**		
GAP responsiveness			5.509	**0.000**
GAP reliability			−8.304	**0.000**
GAP assurance			2.212	**0.000**
GAP tangibles			3.194	**0.001**
GAP empathy			6.785	**0.000**

Highlighted sigma values show statistically significant dimensions.

## Discussion and conclusion

Quality of healthcare has become an increased necessity for patient satisfaction. As observed from this study, steps undertaken in years related to the quality of healthcare in the Albanian public hospital system are very few. However, the situation is changing, and increased attention is being paid to patient opinions and satisfaction by health managers, hospital staff, and policymakers.

The SERVQUAL instrument, selected for the evaluation of patient satisfaction in this study, had its first encounter with the hospital sector in Albania, but it surely proved valuable in determining and evaluating the quality of service and consequently patient satisfaction by meeting the objectives of this paper. From the questionnaire conducted in the 10 largest hospitals in the country located in the main districts, with 800 patients, despite significant improvements made in the last 2 years (as expressed by patients interviewed), there is a long way to be done to enhance the overall level of service quality based on the results of the study. Only 38% of the patients interviewed were very satisfied with the quality of service, while only 29% of them are satisfied. In the public sector, the most valued dimensions are staff assurance and reliability, followed by responsiveness and staff empathy, while tangibles were the worst-rated dimension in this sector.

However, if dimensions of service quality are to be assessed as isolated and evaluated only by patients' real perceptions and the analysis of their relationship with patient satisfaction is measured, the strongest relationship with the satisfaction would be the staff empathy dimension (Table 11), a result to be expected even from the literature (43–45). This dimension has a strong positive correlation with patient satisfaction, showing that if patients perceive staff as polite and that the doctors do genuinely care about them, by giving personal attention, respecting their privacy, and recognizing their specific needs, then patients' satisfaction increases, even if the other elements of the service will falter. Interestingly, this dimension plays a defining role in patient satisfaction that value the empathy of doctors as an even more necessary element than responsiveness and reliability, which shows that sensitivity, courtesy. and being comprehensive toward patients' problems are very important. This result is different from other studies where the most valued dimensions have been tangibles, assurance, and responsibility (46–48).

**Table 11:** Analysis of multiple regression “patient satisfaction—quality of service” at public hospitals based on the real perception of the patients.

**Model**	**Unstandardized coefficients**		***t***	**Sig**.	**95.0% confidence interval for B**		**Collinearity statistics**	
	**B**	**Std. error**			**Lower bound**	**Upper bound**	**Tolerance**	**VIF**
(Constant)	0.746	0.130	−5.749	0.000	−1.001	−0.491		
Responsiveness	**0.143**	0.034	4.251	**0.000**	0.077	0.208	0.393	2.546
Reliability	**0.153**	0.044	3.462	**0.001**	0.066	0.240	0.313	3.199
Assurance	**0.209**	0.045	4.660	**0.000**	0.121	0.298	0.372	2.688
Tangibles	**0.258**	0.031	8.403	**0.000**	0.197	0.318	0.782	1.279
Empathy	**0.393**	0.042	9.347	**0.000**	0.311	0.476	0.280	3.568

Highlighted sigma values show statistically significant dimensions.

Another key element, which is strongly and positively related to patient satisfaction is the responsiveness of the staff where their readiness to help patients and the clear explanation of the procedures and treatments to be performed, would result in satisfied patients as confirmed also by Rezaei et al. (49) and Javed and Ilyas (50). Similarly, as stated by De Man (51) and Zun et al. (11), the dimension of reliability has a positive and relatively strong correlation with patient satisfaction, which increases if the staff devotes the necessary time to the examinations, provides the right service from the first time, and they show honest interest for solving patients' problems.

The assurance which is mostly related to theoretical and technical skills and knowledge of the staff, which results in a better diagnosis of the patients and the accuracy of the laboratory results and the immediate delivery of the emergency service, also turns out to be positively related to the patient's satisfaction, which is also confirmed by Khasimah and Njau (52, 53). The safer they feel about the service received and the way it is delivered, the more satisfied they are with the quality of the hospital service.

However, different to Alghamdi (43) and Baia et al. (54), the poorest relation with patient satisfaction is the dimension of tangibles, which is probably because loyal patients of public hospitals are already aware of the quality and conditions they will find in these hospitals, a result that is validated from Khamis et al. (53). However, improving the conditions and modernizing the equipment would increase their satisfaction. In the public sector, patient expectations were not high and varied from dimension to dimension. The highest expectations (where over 55% of patients expected the situation to be very good) were based on the dimension of assurance that staff creates for patients (and it should be noted that this is the most valued and realistically assessed dimension), followed by reliability, responsiveness, empathy, and finally the dimension with the lowest expectations is again tangibles (where 10% of respondents expected the situation to be very good and almost over 40% of them expected the situation to be at least to the average level).

In terms of expectations they had, patients can be divided into three groups:

- Those who had previously been admitted to the hospital and were aware of the conditions, and consequently their expectations matched their realistic assessment of quality.
- Those patients who expected a very bad condition of the hospitals were influenced by the opinions heard by others or comments from various media, which could then be surprised by the real condition of the hospitals, making the ratings higher than their expectations, and consequently to be more satisfied.
- Patients who expected the hospital condition to be very good, influenced by the latest policies implemented in the country, where their expectations were very high, but after receiving the service, their assessments were medium or low leading to a negative gap and consequently to dissatisfaction.

## Recommendations

The analysis performed and the conclusions obtained show the reality of the situation of the health system in Albania, on its service quality, and patient's perspectives. Despite the fact that in recent years, attention has begun to be paid to quality as a determinant of patient satisfaction, the study and analysis of these elements are still in the first stages. Although patients' assessments of quality are not high in the public sector, according to the analysis performed with the SERVQUAL instrument, they are somehow satisfied. This is because the expectations in this sector for most of the interviewers' have been low or average.

However, what should be emphasized and taken into account is that the public health sector in Albania, in terms of quality of service, barely manages to meet the expectations of patients, even in certain dimensions, although these expectations are very low, still does not reach to meet them.

It is therefore necessary to take into consideration the following recommendations:

1. Hospital managers should conduct periodic surveys through various questionnaires, or other methods to assess service quality, in public hospitals, to be informed about the weak points of the quality of service provided by their hospitals, based on the dimensions of service quality used to assess patient satisfaction.
2. Since the dimension of quality, which has the greatest impact on patient satisfaction, is staff empathy, attention should be paid mainly to staff behavior, conducting various trainings or seminars on how to communicate and act with patients.
3. A review of financial resources and their allocation is needed to increase investments in the technology used and hospital equipment, as this is the dimension that is less valued by patients. A modernization of the technology used for both the diagnosis and treatment of diseases is required. It is also necessary to review the hygiene conditions in hospitals, perhaps by delegating some of the services to the private sector, to increase the quality of service, and to always monitor even the delegated services.
4. Given that the strongest point of the public health sector is related to the professional skills of doctors and support staff, it is crucial to maintain professionalism, by providing doctors with the necessary conditions for further qualifications, as well as supporting them with offering appropriate professional accreditations programs for both staff and hospital service.
5. A very important aspect, which greatly affects the satisfaction of patients is the swiftness of service received. For this reason, the emergency service should be re-evaluated, appointing very specialized staff, who should provide a service not only fast but also effective.
6. It is also important for staff to communicate with patients about the treatments and medications they receive, as this would increase patients' satisfaction with the services provided. To this end, another important fact that needs to be paid attention to by hospital managers is the awareness of staff on informing patients concisely on issues related to their treatment and the medications to be received.

## Limitations of the study

Given that, in Albania, quality measurements of hospital services performed with this instrument or other similar instruments are missing, it is difficult to make a comparative analysis of the results obtained. No matter how large the sampling is, again this number does not represent all patients who have received hospital service, in all surveyed hospitals. There is no certainty about the accuracy and sincerity of the responses received from patients, as most of them were interviewed within the hospital premises, causing patients to be influenced in responding in the presence of the staff or to be reluctant to respond. This is because a large part of the patients refused to be part of this questionnaire, perceiving that it was directed by the hospital managers or media. The 2 months duration of the questionnaire was conditioned by personal factors as well as by the events that occurred within this period.

## Data availability statement

The original contributions presented in the study are included in the article/supplementary material, further inquiries can be directed to the corresponding author/s.

## Ethics statement

Ethical review and approval were not required for the study on human participants in accordance with the local legislation and institutional requirements. Written informed consent from the patients was not required to participate in this study in accordance with the national legislation and the institutional requirements.

## Author contributions

RK: Methodology, Analyses and results, and Discussion and conclusions. MK: Introduction, Literature review, and Recommendations. All authors listed have made a substantial, direct, and intellectual contribution to the work and approved it for publication.
